# Interactions between sympatric invasive European fire ants (*Myrmica rubra*) and blacklegged ticks (*Ixodes scapularis*)

**DOI:** 10.1371/journal.pone.0251497

**Published:** 2021-05-10

**Authors:** Lucy D. Guarnieri, Sara E. McBride, Eleanor Groden, Allison M. Gardner

**Affiliations:** School of Biology and Ecology, University of Maine, Orono, Maine, United States of America; Tufts University Cummings School of Veterinary Medicine, UNITED STATES

## Abstract

The blacklegged tick (*Ixodes scapularis*) and the invasive European fire ant (*Myrmica rubra*) are both expanding throughout their sympatric range in coastal New England. *Ixodes scapularis* is the primary vector of the bacterium *Borrelia burgdorferi*, which is the causative agent of Lyme disease, and Mount Desert Island, Maine, home to Acadia National Park, currently is affected by a high Lyme disease burden. Ticks have many natural predators, including ants, although no previous studies have investigated interactions between these two species. To test the hypothesis that the presence of *M*. *rubra* alters *I*. *scapularis* abundance, we collected ticks by drag-sampling at eight ant-infested sites and eight uninfested control sites in Acadia National Park. We found that nymph density was significantly higher at ant-infested sites, while larval density was significantly higher at control sites. In addition, we conducted a laboratory bioassay to measure *M*. *rubra* aggression against *I*. *scapularis* larvae, nymphs, and adults and *Dermacentor variabilis* adults, and found that ant aggression was significantly higher against *D*. *variabilis* adults than *I*. *scapularis* adults. Our findings support the hypothesis that *M*. *rubra* has divergent effects across *I*. *scapularis* life stages, and we discuss possible ecological mechanisms, including optimal microclimate and predation, that could promote density of nymphs while inhibiting density of larvae.

## Introduction

Invasive species are transforming ecosystems worldwide at an unprecedented scale with negative outcomes for biodiversity [1–3], animal and human health [4], agriculture [5], and forestry [6]. Invasive species interact with sympatric native species through a variety of mechanistic pathways [7, 8]. In the case of invasive or geographically expanding disease vectors, these interspecific interactions may have important impacts on the ecology of vector-borne infectious disease and human and wildlife health [4]. For example, two invasive mosquito disease vectors, *Aedes aegypti* and *Aedes albopictus*, limit each other by competing for nutritional resources in aquatic habitats within their introduced range [9–11], while ticks and mosquitoes may be facilitated by the environmental conditions created by certain invasive plants [12–14] or forest pathogens [15]. However, interactions between co-occurring invasive arthropod disease vectors and expanding non-vector arthropods and the potential consequences for human and wildlife health remain underexplored.

The blacklegged tick, *Ixodes scapularis* (Acari: Ixodidae), is the most medically significant disease vector in North America, transmitting the causative agent of Lyme disease, *Borrelia burgdorferi*, as well as other parasites that cause disease in humans. *Ixodes scapularis* populations have become re-established across the northeastern and upper midwestern U.S. throughout the 21st century [16], reclaiming their original range [17]. This expansion and concomitant increased incidence of tick-borne disease in humans may be due to a combination of climate and habitat changes facilitating *I*. *scapularis* and the vertebrate host species it parasitizes [18–20]. In its northern range, *I*. *scapularis* shares deciduous forest habitat with another invasive arthropod, the European fire ant, *Myrmica rubra* (Hymenoptera: Formicidae). *Myrmica rubra* was accidentally introduced to North America from Europe in the early 1900s, and, facilitated by climate change and transportation of soil and plant material, has become established and is anticipated to continue to spread throughout coastal New England [21, 22]. Ecological and economic consequences of *M*. *rubra* infestation include loss of native fauna [23, 24], steep declines in native ant diversity [25, 26], reduced property value, and human exposure to painful stings.

*Myrmica rubra* could either inhibit or facilitate *I*. *scapularis* and the pathogens it transmits by a variety of mechanisms. Ants have been cited as predators of ticks more than any other arthropod group in laboratory and field studies [27]. Multiple field studies indicate that the density of nymphs (DON) may be suppressed by the presence and/or density of nests of two invasive ant species, the imported fire ant, *Solenopsis invicta*, and the European red wood ant, *Formica polyctena* [28, 29]. Although *M*. *rubra* is omnivorous [30–32], it may have similar impacts on *I*. *scapularis* populations. Alternatively, if *M*. *rubra* is not itself a natural predator of ticks, it may have the opposite effect of *facilitating* survival of *I*. *scapularis* indirectly by suppressing populations of native arthropod predators of ticks [27, 33], especially native ants [26, 31].

In this study, we investigated the associations between *M*. *rubra* infestation and *I*. *scapularis* density and infection prevalence to understand the interactions between these two sympatric expanding species and their potential epidemiological consequences. First, we conducted a field study to test the hypotheses that density of larval and nymphal ticks are different in *M*. *rubra*-infested areas compared to uninfested areas. We also conducted supporting laboratory experiments to test the hypothesis that *M*. *rubra* preys upon one or more life stages of *I*. *scapularis* and a second highly abundant tick species in Maine, the American dog tick, *Dermacentor variabilis*.

## Materials and methods

### Study site selection

**Fig 1:**
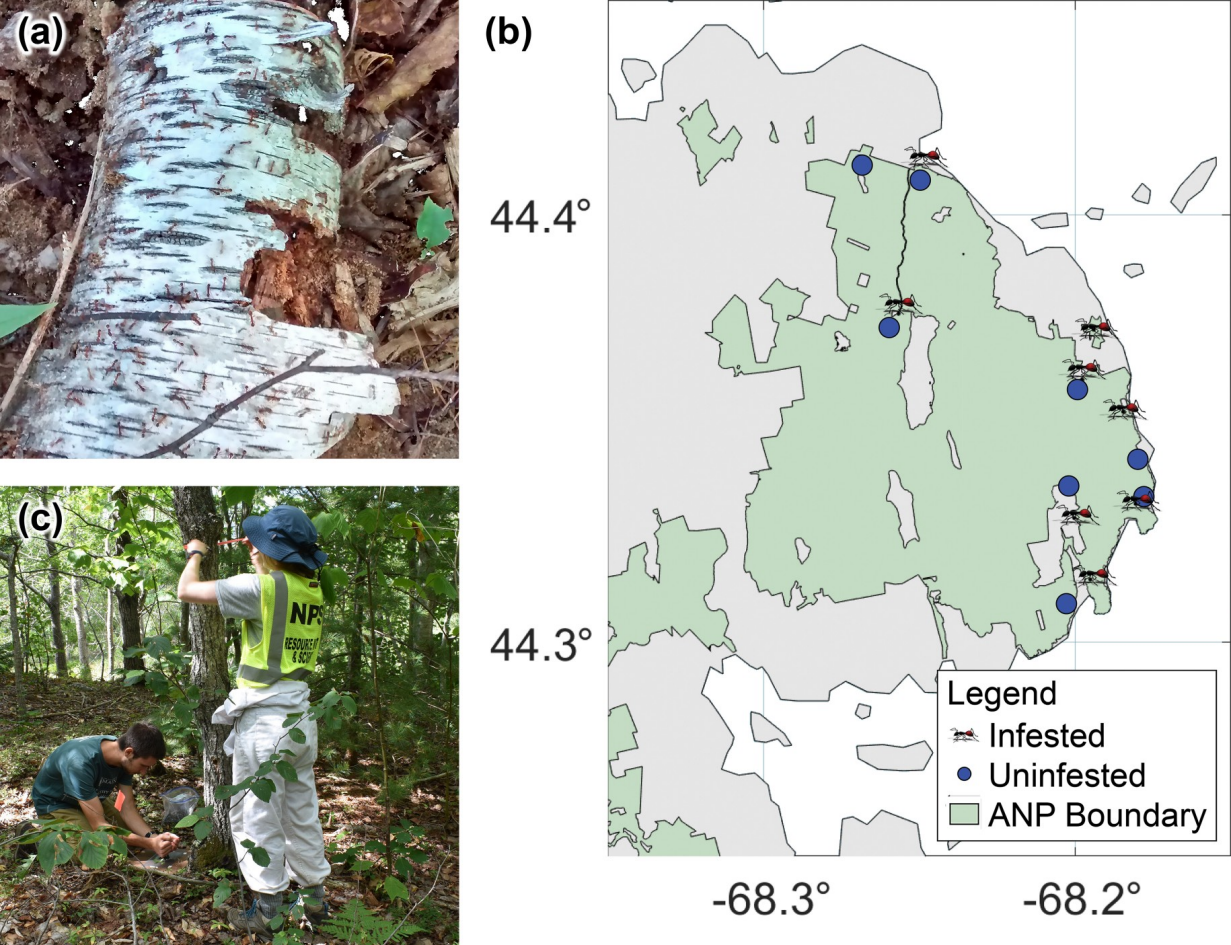
Photograph of *Myrmica rubra* infestation (a) and map of spatial configuration of *M*. *rubra*-infested and uninfested control study sites in Acadia National Park (b). Each site was characterized by deciduous or mixed canopy cover with leaf litter and perennial undergrowth (c).

Field work was conducted in Acadia National Park, Mount Desert Island (MDI), Maine, a popular tourist destination visited by ~3.5M people annually during the summer and early fall when ticks are active [34]. Field research was conducted under scientific research and collecting permit number ACAD-2019-SCI-0007 granted to AMG by the National Park Service. Eight *M*. *rubra* infestations of varying size and shape were selected as treatment sites. *Myrmica rubra* had been established at these sites for at least 15 years [21] as of the beginning of the study. Visual surveys were conducted to determine the presence or absence of *M*. *rubra*, which is readily distinguished from native ant species on MDI by their extremely high nest densities (~1.24 nests/m^2^, Fig 1A) [21]. To account for fine-scale habitat variation on MDI, including soil type, leaf litter depth, and other unmeasured or latent variables, each treatment site was paired to a control site that was adjacent if possible, and otherwise within 7.5 km (Fig 1B). The mean distance between paired sites was 1.4 ± 2.3 km. All sites were in deciduous or mixed forest with leaf litter and perennial undergrowth (Fig 1C), were adjacent to roads, and ranged approximately from 1,000–4,500 m^2^ in area. Because elevation is a predictor of humidity, temperature, and *I*. *scapularis* density on MDI [35], to further control for fine-scale microclimate variation, the elevation at the center of each site was determined using Google Earth and elevation was included as a covariate in statistical analyses.

### Tick collection

To test the hypothesis that *M*. *rubra* presence alters *I*. *scapularis* abundance, we collected ticks via drag sampling [36], whereby a 1 m^2^ square cloth attached to a wooden rod is pulled across the ground to pick up questing ticks. Dragging was conducted for a period of one person-hour per site and tick density was estimated as number of ticks collected per hour. During dragging, the cloth was inspected for ticks approximately every 30 seconds. Nymphs were transferred into Eppendorf tubes with 70% ethanol. Larvae were removed with a lint roller at the end of the drag session and the lint sheets were stored in Ziploc bags. Ticks were identified to species using dichotomous keys [37].

Drag sampling took place in mid-July and late August, 2018, to capture peak activity periods of *I*. *scapularis* nymphs and larvae, respectively. Paired sites were sampled on the same day in arbitrary order. All sampling was conducted between the hours of 0900 and 1600 under clear weather conditions with temperatures between 22–33°C. Tick abundance data were visualized in R version 4.0.0 [38] using the R package ggplot2 [39]. We carried out separate generalized linear mixed effect models using the R package lme4 [40] to compare nymphal and larval tick abundance, modeled as Poisson distributions, across treatment (i.e., infested or uninfested by *M*. *rubra*), month of collection, and elevation (m). *Myrmica rubra* infestation was treated as a binary response variable (i.e., present or absent). Site pair was included as a random effect. Goodness of fit was determined by calculating pseudo-R^2^ using the package sjstats [41].

### Aggression bioassay

**Fig 2:**
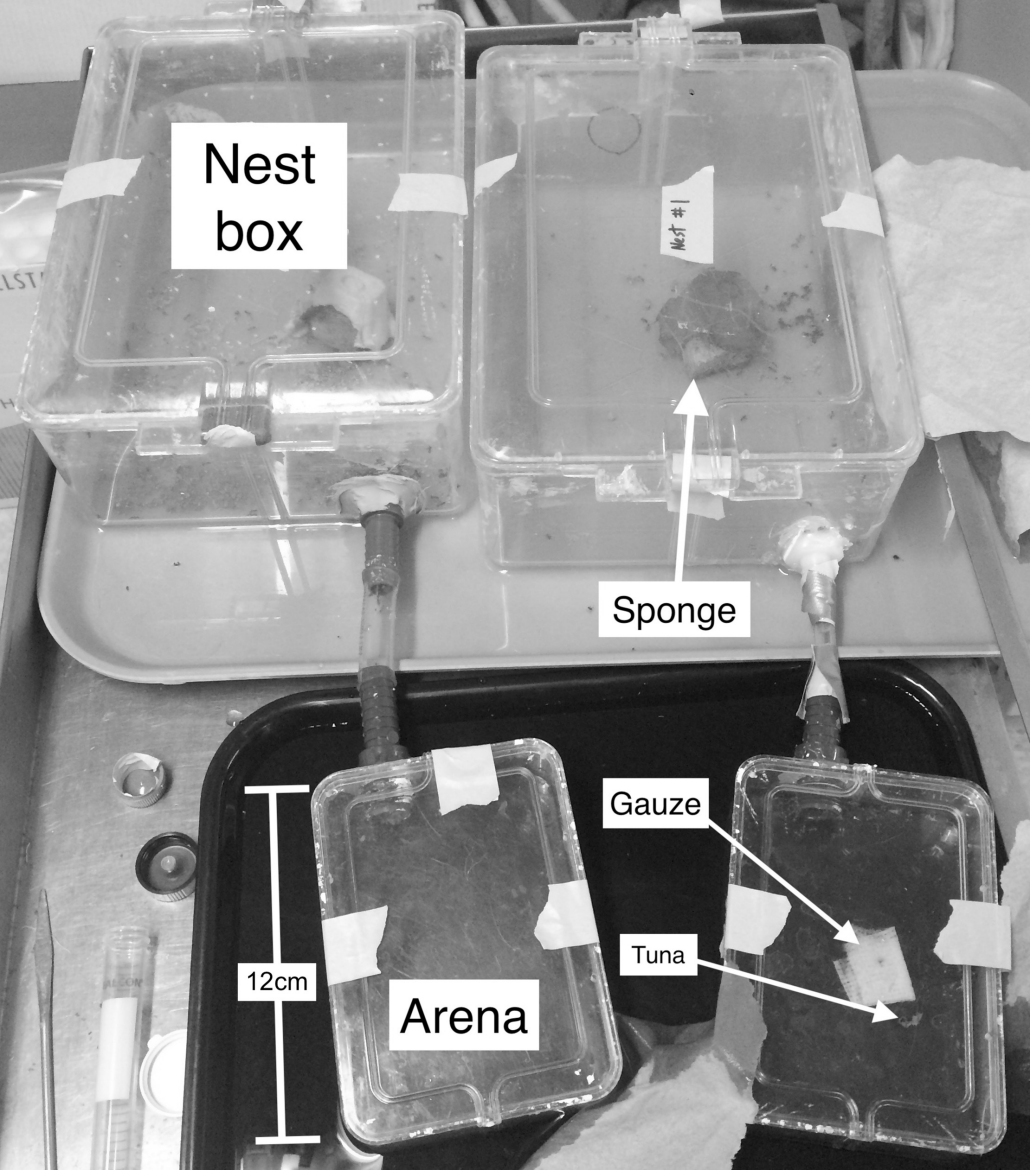
Ant nest boxes with connected arenas used to conduct aggression bioassays. Ant nests were maintained in nest boxes and *Myrmica rubra* were allowed to interact with *Ixodes scapularis* larvae, nymphs, and adults and *Dermacentor variabilis* adults.

To test the hypothesis that *M*. *rubra* preys on one or more *I*. *scapularis* life stages, we conducted a laboratory aggression assay modeled upon the methods of Garnas et al. [42]. Two *M*. *rubra* nests were collected in Orono, ME (44.88°N, 68.66) for use in the assay. Each nest contained workers, queens and brood. Nests were housed in separate 9.8-liter plastic boxes connected with tubing to smaller arenas (Fig 2). The inside walls of the enclosures were coated with Fluon to prevent ants from climbing. Ants were fed with frozen tuna and gauze soaked in 25% sucrose solution every two days and starved for at least 48 hours before use in a trial. Moisture was provided periodically by pipetting water onto a 3 cm^3^ piece of sponge.

We tested *M*. *rubra* aggression against *I*. *scapularis* adults, nymphs, and larvae and adult *D*. *variabilis*. Adult *D*. *variabilis* were included to serve as an outgroup because, as a larger and more mobile species, we hypothesized they may elicit different aggressive behavior from *M*. *rubra*. Lab-reared ticks were obtained from the Oklahoma State University Tick Rearing Facility (Stillwater, OK) to minimize risk of investigator exposure to tick-borne pathogens. All ticks were unfed. Before each trial, we removed ants from the arena, blocked the entrance, then wiped and dried the inside surfaces with a damp sponge and paper towel to remove any pheromones from previous trials. After gently replacing the ants inside the arena, we unblocked the entrance, allowing the ants to move freely between the nest box and the arena. A few minutes later, we placed either three adults, three nymphs, or six larvae in the center of the arena. Larvae were tested in groups of six due to their relatively small size and low rate of encounter by the ants. We tallied the following ant behaviors over 10 minutes: antennation (probing tick with antennae), threat (lunging with mandibles open), biting, carrying/dragging, and stinging. We tested each set of ticks five times for a total of 20 trials, and calculated aggression scores according to [42], with an equation modeled after De Vroey and Pasteels [43]: Score = (1 x threats) + (2 x bites) + (2 x carrying) + (3 x stings). We carried out a generalized linear model using the package MASS [44] to compare aggression score, modeled as a Poisson distribution, across tick species and nests.

## Results

**Fig 3:**
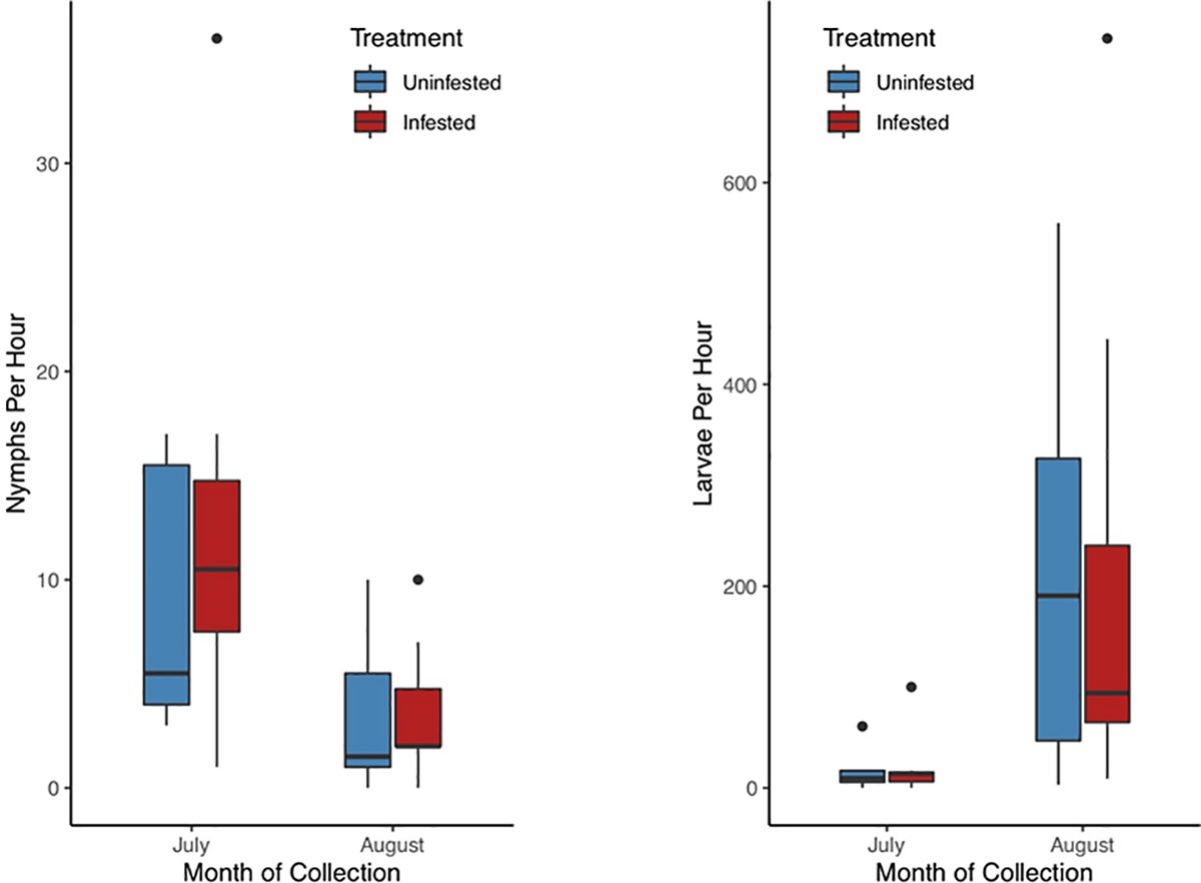
Comparison of (a) nymphal and (b) larval *Ixodes scapularis* density at *Myrmica rubra*-infested and uninfested study sites.

We collected a total of 232 *I*. *scapularis* nymphs and 3,778 *I*. *scapularis* larvae throughout the field study (Fig 3A and 3B). No adult ticks were encountered. Nymphal abundance was higher in *M*. *rubra-*infested areas compared to uninfested areas (Z = 2.61, P < 0.01, pseudo-R^2^ = 0.77), while larval abundance was lower in ant-infested areas compared to control areas (Z = -9.76, P < 0.01, pseudo-R^2^ = 0.98). As predicted based on the *I*. *scapularis* life cycle, nymphal abundance was higher (Z = 7.28, P < 0.01) and larval abundance was lower (Z = -40.73, P < 0.01) in July compared to August. There was no significant relationship between elevation and nymphal density (Z = 1.60, P = 0.11), although elevation was negatively associated with larval density (Z = -11.60, P < 0.01).

**Fig 4:**
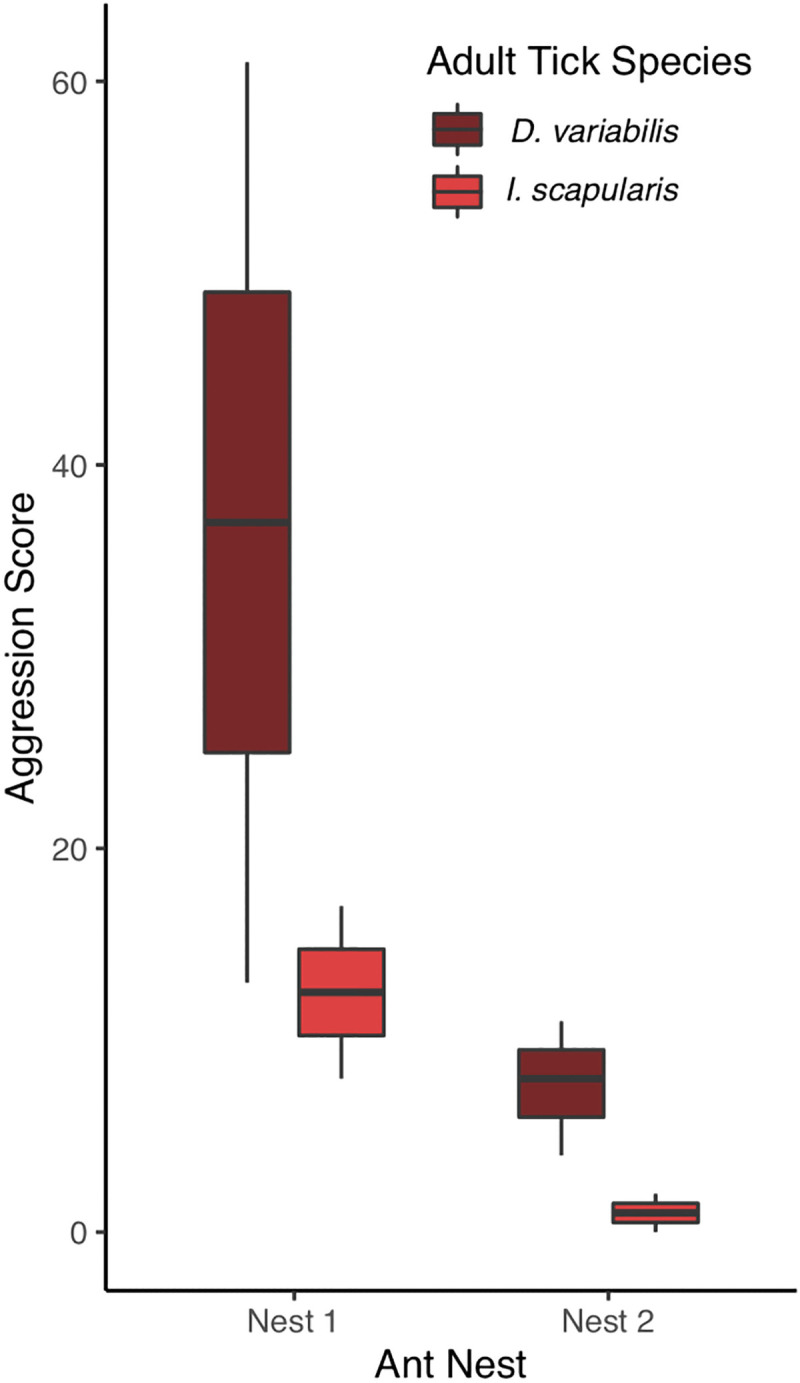
*Myrmica rubra* aggression scores against unfed adult ticks by tick species and by ant nest. No aggression was documented against nymphs or larvae.

In laboratory bioassays, *M*. *rubra* displayed greater aggression toward D. *variabilis* adults than toward *I*. *scapularis* adults (Z = 5.79, P < 0.001), and overall, aggression was higher within Nest 1 than within Nest 2 (Z = 7.91, P < 0.001; Fig 4). The mean aggression score for *I*. *scapularis* adults was 5.6 and the mean score for *D*. *variabilis* adults was 19.4. *Myrmica rubra* never displayed aggressive behavior towards nymphs and larvae; therefore, scores for these life stages were always zeros.

## Discussion

There is substantial evidence that invasive species may inhibit or facilitate native species [8]; in the case of geographically expanding arthropod disease vectors, the outcomes of these interactions may be highly consequential for human and animal health. This research effort combined field and laboratory studies to explore associations between the blacklegged tick, *I*. *scapularis*, and the invasive European fire ant, *M*. *rubra*, two human health and economic pest species that co-occur in coastal New England and are expanding in geographic range. We found that *I*. *scapularis* nymph density was significantly higher, and larval density significantly lower, at *M*. *rubra* infested sites compared to paired uninfested sites in Acadia National Park. We also found that *M*. *rubra* do not behave aggressively toward unfed *I*. *scapularis* nymphs or larvae under laboratory conditions, and that *M*. *rubra* are significantly more aggressive toward unfed adult American dog ticks, *D*. *variabilis*, compared to adult *I*. *scapularis*. Collectively, our findings support the hypothesis that *M*. *rubra* predation has different associations with the densities of *I*. *scapularis* nymphs and larvae.

Contrary to previous studies that have demonstrated negative associations between the presence of the invasive ants *Solenopsis invicta* and *Formica polyctena* and the abundance of hard-bodied tick species [28, 29], our field studies suggest that *M*. *rubra* presence is *positively* associated with *I*. *scapularis* nymph abundance. Positive correlation between nymph abundance and ant presence could be attributed to similar preferred habitat. *Myrmica rubra* arrived on MDI roughly 50 years ago and since have spread following a jump dispersal pattern [21]. While the recent spread of *I*. *scapularis* across the entire island [35] occurred independently of *M*. *rubra*, both species’ survival is enhanced by conditions in deciduous habitats and therefore their densities likely are correlated. Landscape features, soil types, microclimate conditions, and nymph densities vary significantly across ANP [35, 45]. While we designed our field study to control for unmeasured variables that may impact questing ticks, fine-scale microhabitat variation nonetheless may have contributed to our findings. *Myrmica rubra* also may facilitate *I*. *scapularis*; myriad examples of such facilitation of arthropod disease vectors by either native or non-native plant and insect species have been documented previously [46–48]. As soil engineers, *M*. *rubra* increase soil aeration and drainage [49, 50], which may create a favorable soil microenvironment for *I*. *scapularis*. Given that richness and diversity of native ant fauna are significantly reduced by *M*. *rubra* [26, 31] and native ants are natural predators of *I*. *scapularis* [27], the tick could also benefit from displacement of predaceous ants from *M*. *rubra*-infested areas. Unlike nymphs, which were distributed relatively homogeneously throughout sites, larvae were clustered in patches, and thus larval density was highly variable, with counts ranging from 0–560 larvae per hour for uninfested sites and 0–743 for *M*. *rubra* sites. Interestingly, our analysis also indicates a significant negative association between *M*. *rubra* presence and *I*. *scapularis* larval density. Future research should seek to identify mechanistic drivers of this mismatch in tick densities across life stages, potentially including impacts of *M*. *rubra* on activity of key blood-meal hosts for adult *I*. *scapularis*, e.g., white-tailed deer [51] and predation of *I*. *scapularis* eggs or larvae in the soil [52, 53].

*Myrmica rubra* never displayed aggressive behavior (i.e., threatening, biting, carrying, stinging) towards nymphs or larvae in our laboratory bioassay. In general, nymphs and larvae moved more slowly than adult ticks and were also more likely to be entirely immobile; thus, juvenile ticks may have appeared less threatening to *M*. *rubra* than adults. In addition, larger ticks may be perceived as more valuable prey; the sclerotized, chitinous exoskeleton of a hard-bodied tick is a formidable barrier for small predators like ants [54], and smaller ticks offer less reward for the effort, as indicated by increased rates of predation by several species of ants on engorged ticks compared to flat ticks [27]. Heightened aggression toward *D*. *variabilis* compared to *I*. *scapularis* may be explained by several mechanisms. Adult *D*. *variabilis* are up to twice as long as *I*. *scapularis*, and *D*. *variabilis* often flipped onto their backs and waved their legs in the air during the bioassay; these differences in behavior and morphology could lead to the perception of *D*. *variabilis* as more threatening and/or more valuable prey to *M*. *rubra*. In addition, many Ixodid species secrete an allomone that masks their presence from predaceous ants [55–57], though the efficacy of such secretions under laboratory conditions is unknown.

Although unexplored in this study, *M*. *rubra* infestation also may impact the proportion of ticks infected with *B*. *burgdorferi* and other tick-borne pathogens. Nymphal infection prevalence largely reflects the availability of competent pathogen reservoir hosts, such as the white-footed mouse, *Peromyscus leucopus* [58], to host-seeking ticks. Stinging ants may reduce small mammal abundance [59] and cause changes to small mammal behavior, such as increased foraging efficiency [60], thereby lowering tick-host encounter frequencies and reducing nymphal infection prevalence. *Myrmica rubra* also may alter the diversity of small mammal hosts [61], alternately weakening or strengthening the dilution effect, wherein incompetent pathogen reservoirs deflect blood meals from competent hosts [62], potentially affecting nymphal infection prevalence. The effects of *M*. *rubra* on pathogen prevalence should be investigated in future research due to its implications for human health.

## Conclusion

*Myrmica rubra* causes significant loss of native biodiversity as well as property value in its invaded range, and *I*. *scapularis* poses serious threats to human and animal health. Here, we investigated associations between *M*. *rubra* presence and *I*. *scapularis* nymph and larval density within their sympatric range. We found that *M*. *rubra*-infested sites had higher densities of *I*. *scapularis* nymphs and lower densities of *I*. *scapularis* larvae. Mechanisms to explain this result may include differences in optimal habitat between the life stages and/or ant predation on engorged females or eggs. This study presents new evidence for associations between *M*. *rubra* presence and *I*. *scapularis* nymphal and larval densities, highlighting the importance of researching interactions between sympatric expanding species and the implications for vector borne disease.

## Supporting information

S1 Data (CSV)

S2 Data (CSV)
